# Antidepressant-Induced Apathy in Adolescents with a Depressive Episode While Taking Sertraline: Results of 8-Week Observational Study with Pharmacogenetic Testing for *CYP2C19*

**DOI:** 10.3390/biomedicines14030735

**Published:** 2026-03-23

**Authors:** Dmitriy V. Ivashchenko, Sergey V. Grass, Vitaliy V. Sobur, Anna Y. Basova, Pavel V. Shimanov, Artem V. Shubin, Roman V. Deitch, Svetlana N. Tuchkova, Ivan N. Korsakov, Karin B. Mirzaev, Yuriy S. Shevchenko, Dmitry A. Sychev

**Affiliations:** 1 Federal State Budgetary Research Institution «Russian Research Center of Surgery Named After Academician B.V. Petrovsky», 119435 Moscow, Russiakarin05doc@yandex.ru (K.B.M.); dmitry.alex.sychev@gmail.com (D.A.S.); 2 Russian Medical Academy of Continuous Professional Education, 125993 Moscow, Russia; 3 Scientific-Practical Children’s and Adolescents Mental Health Center n.a. G.E. Sukhareva, 119334 Moscow, Russia

**Keywords:** gene, genome, genetics, genotype, depression, sertraline, apathy, emotional blunting, adverse effect, safety, adolescents, CYP2C19

## Abstract

**Objectives**. The aim of our study was to track changes in ODQ scores in adolescents with depressive episodes taking sertraline, depending on *CYP2C19* polymorphisms. **Methods**. This study included 88 adolescents (88% were female) aged 12–17 who were prescribed sertraline. Emotional blunting was assessed using the Oxford Depression Questionnaire (ODQ) scale when the antidepressant was prescribed, after one, three, and 8 weeks, taking into account other medications used. Part 3 of the ODQ scale assessed the changes that occurred after the prescription of an antidepressant. All patients were genotyped for CYP2C19*2, *3, and *17. Based on genotypes, the phenotypes of the CYP2C19 isoenzyme were determined. **Results**. The ODQ score at the time of enrollment was higher (65[50;79] points) compared with after 8 weeks (38.5[32.5;56.5] points). Part 3 of the ODQ-26 questionnaire remained approximately the same for 8 weeks. Patients with higher ODQ-26 values at enrollment (73[56;83] vs. 59[44;71] points) were more likely to be prescribed antipsychotics. Differences in ODQ scores remained significant up to 3 weeks after enrollment (50.5[41.5;68] vs. 45.5[36;54] points). The comparison of ODQ scores and their dynamics did not show significant differences depending on CYP2C19*2 or *17 polymorphisms, or the type of CYP2C19 metabolism. **Conclusions**. There was no increase in emotional blunting according to the ODQ score among adolescents with depression who took sertraline for eight weeks. No significant correlations were found between the carrier status of *CYP2C19* gene variants and the development of apathy induced by antidepressants.

## 1. Introduction

Apathy, one of the manifestations of depression, represents a lack of motivation that is not associated with impaired cognitive function, consciousness, or emotional stress [1]. Apathy is one of the manifestations of depressive syndrome. “Apathy” or “emotional blunting” is also a side-effect of selective serotonin reuptake inhibitors (SSRIs) and other monoaminergic antidepressants [2]. But when we talk about emotional blunting, we are referring specifically to changes that have occurred as a result of taking antidepressants. This was first shown in a study by Opbroek et al. (2002): patients noted a significant narrowing of the emotional range experienced when taking SSRIs [3]. According to patient surveys, the feeling of “apathy” when taking SSRIs differs from the manifestations of a depressive episode [2]. This undesirable reaction is observed when taking all monoaminergic antidepressants [4]. SSRI-induced apathy manifests itself as a loss of initiative and indifference to others and is more pronounced with a greater severity of the depressive episode [4]. The situation is further complicated by the fact that the very nature of a depressive episode involves apathy. The patient must determine whether the emotional blunting occurred as a result of taking the antidepressant or whether this symptom was present from the beginning. While taking antidepressants, mood improves, and the patient may be better able to assess their condition, noticing new symptoms. This view is reflected in the study by Peters et al. (2022): complaints of apathy during the first few weeks of antidepressant treatment may be a residual symptom of a depressive episode [5]. However, this issue requires further study using specially designed assessment tools, as it has been demonstrated that emotional numbness can persist for weeks or even months [4].

Today, antidepressant-induced emotional blunting has been well studied among adult patients [6]. This is a dose-dependent, reversible adverse reaction observed in 20–92% of patients who take SSRIs [6]. According to the conducted research, emotional dullness becomes one of the reasons for discontinuation of SSRIs [7,8]. The neurobiology of antidepressant-induced emotional blunting has not been sufficiently studied to date [9,10]. There is a hypothesis that SSRIs increase serotonin levels, which, in turn, increases the activity of GABA-ergic interneurons. Emotional blunting occurs as a phenotypic manifestation of decreased dopamine and norepinephrine levels in the central nervous system [10,11]. Taking SSRIs leads to emotional blunting indirectly, through a complex system of neurotransmitters, the main actors being dopamine and norepinephrine. The effect is dose-dependent; therefore, the concentration of the antidepressant in the blood is important [11].

A special scale, the Oxford Depression Questionnaire, has been developed to assess emotional blunting [7,12]. The scale contains 26 questions, of which the last six (Part 3) are to be answered by patients taking an antidepressant [12]. The use of the ODQ in adult patients with a depressive episode makes it possible to successfully assess the degree of emotional blunting, as well as the dynamics of improvement in the patient’s condition [4,6,7,8]. Apart from two case report series [13,14], there are no studies of antidepressant-induced emotional blunting in adolescents. Emotional indifference has been observed in adolescents taking fluvoxamine, fluoxetine, and paroxetine [13,14]. Studies using the ODQ-26 questionnaire in patients under the age of 18 have not been found in the literature.

It is known that emotional blunting causes premature cancellation of antidepressants and, thus, increases the risk of relapse of a mental disorder [6,8]. Apathy is reversible and even dose-dependent, which allows us to discuss its correction and prevention [6].

In Russia, sertraline is most commonly prescribed to children, as it is approved for use from the age of 6. Other SSRIs (except fluvoxamine) are approved for use from the age of 18 and are, therefore, less commonly used in children.

Pharmacogenetic testing is an effective way to improve the effectiveness and safety of antidepressants [15,16]. Genetic predictors of SSRI response have been discovered for adults [11,13,14], with studies including adolescents showing paradoxical results [17,18]. This suggests the need for new associative pharmacogenetic studies to identify significant biomarkers. Sertraline is primarily metabolized by the CYP2C19 isoenzyme, so carriage of *CYP2C19* gene polymorphisms may affect plasma concentrations of sertraline [19,20]. Since emotional blunting is a dose-dependent adverse reaction, CYP2C19 genetic testing may be useful in predicting the safety of sertraline. No pharmacogenetic studies of sertraline have been conducted to evaluate changes in ODQ scores.

The aim of our study was to track changes in ODQ scores in adolescents with depressive episodes taking sertraline, depending on *CYP2C19* polymorphisms.

## 2. Materials and Methods

### 2.1. General Description of This Study

In our study, we assessed emotional blunting using the ODQ-26 questionnaire in adolescents with a depressive episode and suicidal intentions who were prescribed sertraline. We chose sertraline because in Russia it is most commonly used as first-line therapy in children and adolescents. Firstly, this phenomenon has not been previously studied in detail in this population. Secondly, there are currently no pharmacogenetic studies of developing emotional blunting in either adults or children while taking the antidepressant. Sertraline is primarily metabolized by the CYP2C19 isoenzyme [21]. Therefore, we chose pharmacogenetic testing of polymorphic variants CYP2C19*2, *3, and *17. Their carrier status leads to both a slowdown in the metabolic rate of the isoenzyme (CYP2C19*2, *3) and a significant acceleration (CYP2C19*17) [22]. Therefore, the pharmacogenetically determined sertraline metabolic rate may be related to the severity of emotional blunting in adolescents.

This study was approved by the local ethics committee of the “Scientific-Practical Children’s and Adolescents Mental Health Center n.a. G.E. Sukhareva” (Meeting No. 2/23 dated 17 May 2023).

Study design: Prospective observational. This study involved patients admitted to the “Scientific-Practical Children’s and Adolescents Mental Health Center n.a. G.E. Sukhareva” from 20 May 2023 to 31 August 2024.

The medical records of children admitted to inpatient treatment were examined for compliance with the inclusion and exclusion criteria.

Inclusion criteria:

- Age from 12 to 17 years old inclusive.
- Depressive syndrome was the main reason for treatment.
- Suicidal ideation or attempt.
- Prescription of sertraline.
- Signed voluntary informed parental consent for the patient’s participation in this study.

Exclusion criteria:

- Diagnosis of bipolar affective disorder (F31.X by ICD-10).
- Diagnosis of schizophrenic spectrum disorders (F2X by ICD-10).

As a result of the screening of adolescents who were hospitalized with depressive syndrome, 133 patients were selected.

The inclusion of the patients in this study was carried out on the first day of their admission to the psychiatric hospital. Each patient’s legally authorized representative signed an informed, voluntary consent to participate in this study. This study complied with the Declaration of Helsinki.

### 2.2. Sample Clinical and Demographic Characteristics

The individual registration chart for each patient included gender, age, height, weight, body mass index, main diagnosis, total number of hospitalizations, age of onset of symptoms of the mental disorder, history of a suicide attempt, having performed non-suicidal self-harm, age of occurrence of non-suicidal self-harm (if any), age of the first suicide attempt (if available), the total number of suicidal attempts (if available), the presence of suicidal thoughts at the time of examination, and having taken an antidepressant immediately before hospitalization.

### 2.3. Assessment of Emotional Blunting Using the ODQ Scale

During the dynamic follow-up, all patients underwent an assessment of the degree of emotional blunting on the Oxford Depression Questionnaire scale (ODQ-26). The ODQ contains 26 questions, 20 of which can be asked of the patient before starting antidepressant treatment to assess the initial severity of emotional blunting. The last 6 questions of the ODQ (Part 3) are only for patients who are already taking antidepressants. These questions help assess changes in emotional state that have happened since starting antidepressants. At the time of inclusion in this study, the patient filled out only the first two parts of the questionnaire (ODQ-20), while upon examination after 1 week, 3 weeks and 8 weeks, the patient filled out the full version: ODQ-26. As a result, a total ODQ score was obtained for each patient. The number of points received when completing the third part of the ODQ questionnaire (changes that occurred after the appointment of an antidepressant) was taken into account separately after one, three and eight weeks.

In addition, a survey was conducted using the Beck Depression Inventory [23], assessment of the severity of the patient’s condition using Clinical Global Impression—Severity, Improvement (CGI-S, CGI-I) [24].

### 2.4. Pharmacotherapy

The researcher could not influence the appointment of psychopharmacotherapy by the attending physician. All psychotropic drugs with daily dosages received by the patient were entered into an individual registration card.

All patients received sertraline as their primary therapy. The starting dose of sertraline was usually 25 mg/day. Subsequently, the treating physician increased the dosage based on the tolerability of the therapy. The standard titration regimen was to increase the dose by 25 mg/day weekly; sometimes, the dosage was increased more slowly. When assessing the patient’s condition at the time of enrollment in this study, after 1 and 3 weeks, the investigator entered the dose of sertraline at the time of examination in the registration chart. In our sample, there were no cases of sertraline being discontinued or replaced with another antidepressant. Some patients were additionally prescribed antipsychotics, mood stabilizers, anticholinergic drugs to correct extrapyramidal symptoms, and anxiolytics. Such cases were considered as polypharmacy and were necessarily taken into account in the analysis.

### 2.5. Genotyping

On the day of inclusion in this study, 5 mL of blood was collected from each patient in test tubes in disposable sterile vacuum tubes with EDTA for the purpose of subsequent genotyping. The blood was taken simultaneously with routine analyses and did not require additional venipunctures. The blood was frozen at −20 °C, transported to the laboratory and subsequently stored at −70 °C.

The laboratory part of this study was conducted at the Research Institute of Molecular and Personalized Medicine of the Russian Medical Academy of Continuous Professional Education (Moscow). DNA isolation and genotyping of the samples took place as they were received between 1 June 2023 and 31 August 2024.

DNA was isolated from venous blood using the column method using the QIAamp DNA Blood Mini Kit (Qiagen, Hilden, Germany). The concentrations and quality assessment of the obtained DNA preparations were carried out using a Qubit 4 fluorimeter (Thermo Fisher Scientific, Waltham, MA, USA) and a Nanodrop ND-1000 spectrophotometer (Thermo Fisher Scientific, Waltham, MA, USA).

Genetic polymorphisms CYP2C19*2 (rs4244285 G681A), CYP2C19*3 (rs4986893 G636A), and CYP2C19*17 (rs12248560 C-806T) were determined by real-time polymerase chain reaction (PCR) using commercial reagent kits (equipment: CFX96 TouchTM Real-Time PCR Detection System (Bio-Rad, Hercules, CA, USA).

All patients were divided into subgroups according to the genotypes of the polymorphisms: carriers of the polymorphic allele (heterozygotes+homozygotes) and homozygotes for the “wild” allele. For example, carriers of the polymorphic variant CYP2C19*2 were divided into two subgroups: GG and GA+AA. Subsequently, the genetically determined subgroups were compared to find associations with the clinical parameters of the patients.

According to the results of pharmacogenetic testing, the type of CYP2C19 metabolism was determined for each patient according to the Dutch Pharmacogenetics Working Group algorithm: “ultrarapid”, “normal”, “intermediate”, and “poor” [25].

### 2.6. Statistical Analysis

Statistical analysis was carried out using SPSS Statistics 26.0. Due to the abnormal distribution of the data, nonparametric criteria were used to compare quantitative variables between groups. The results of calculations of quantitative variables were presented as median and quartiles (Me [Q1; Q3]).

No preliminary sample size calculation was performed. The preliminary sample size was based on the number of patients in previous studies on adverse reactions to antidepressants.

The Mann–Whitney criterion was used to compare the selected subgroups at the same time point according to quantitative variables. The frequencies of categorical variables were compared with each other using Pearson’s chi-square, and Fisher’s exact criterion was used for 2 × 2 comparisons. For repeated measurements, the Wilcoxon matched-pairs test was used for quantitative variables and the McNemar chi-square for categorical variables. Only cases with full data on each time point were analyzed. The primary outcome of this study was the occurrence and severity of emotional blunting. Bonferroni correction was introduced when comparisons of emotional dullness degree were performed. As no statistically significant differences were found, *p*-values were presented without the correction for the readers to be able to assess the statistical significance of differences. The calculation of the correspondence of the genotype distribution to the Hardy–Weinberg law was performed using an online calculator [26].

## 3. Results

This study initially included 133 patients. The clinical and demographic characteristics of the patients are presented in Table 1. No significant differences in baseline characteristics were observed between the total patient population and those who completed this study.

**Figure 1:**
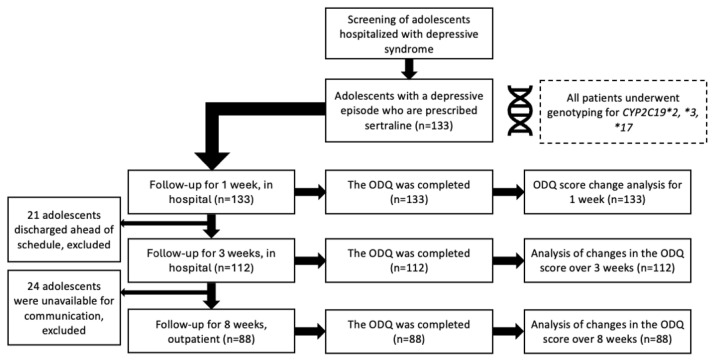
Enrollment and outcomes. Notes: ODQ—Oxford Depression Questionnaire; four patients were excluded before the final analysis due to the absence of ODQ results in the 3rd week.

Figure 1 shows the dynamics of this study.

At the time of inclusion, 102 participants were antidepressant-naïve. However, 21 patients completed the ODQ-26 at the time of admission, as they were taking an antidepressant before hospitalization. On day 7, all 133 patients completed ODQ-26; after 3 weeks, 112 patients completed ODQ-26. At the end of this study (8 weeks), only 88 patients had completed the ODQ-26.

Table 2 shows the results of the ODQ analysis among patients. In the analysis, the total ODQ-26 score was calculated, and the scores of parts 1–2 and the third part of the ODQ, associated with mood changes while taking an antidepressant, were shown separately. As follows from Table 2, there was a significant decrease in the overall ODQ-26 score at week 3 of receiving therapy. During the first week, the value of the ODQ-26 scale score hardly changed. By the 8th week of follow-up, the total ODQ-26 score decreased almost twofold in all patients. 

Then, we compared the ODQ-26 score between carriers of different *CYP2C19* polymorphisms, as well as between “normal” and “intermediate” CYP2C19 metabolizers (Table 2). Among the sample, 28 carriers of the CYP2C19*2 polymorphism and 50 carriers of CYP2C19*17 were identified. Our study did not identify any carriers of the CYP2C19*3 polymorphism, so the comparison was carried out only for carriers of CYP2C19*2 and *17. According to the DPWG algorithm [25], 96 patients had “normal” CYP2C19 metabolism, 27 had “intermediate” metabolism, and eight had “ultrarapid” metabolism. Only two patients had a “poor” metabolism. The “ultrarapid” and “poor” metabolizers were excluded from the comparative analysis. At the time of inclusion, few differences were identified between carriers of the CYP2C19*2 polymorphism on the CGI-S scale (*p* = 0.042), as well as between “poor” and “intermediate” metabolizers on parts 1–2 of the ODQ scale (*p* = 0.028). However, during repeated visits during the observation period, the differences were leveled out.

A comparison of other scales (CGI-S, CGI-I, and BDI) used to assess the patient’s mental state also yielded negative results.

**Figure 2:**
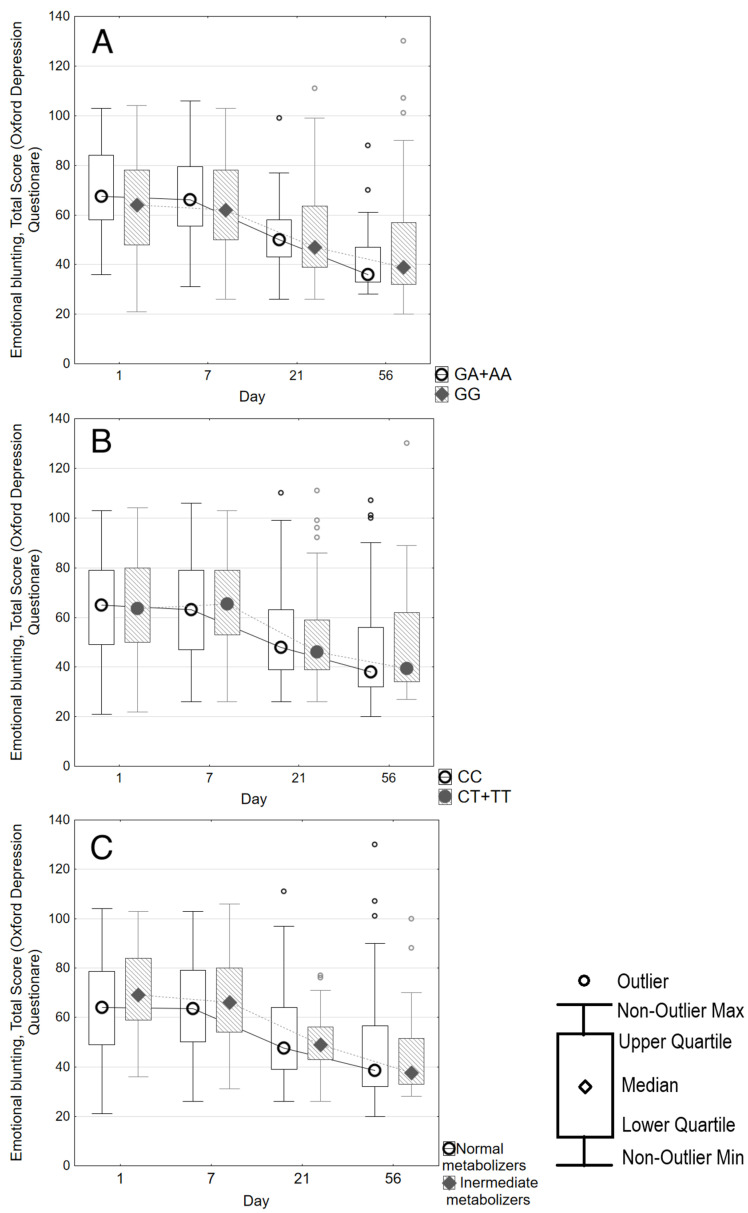
ODQ-26 score changes over 8 weeks according to CYP2C19 genotype. Part (**A**) stratifies the score into wild type (n = 105) or genotype *2 (n = 28). Part (**B**) stratifies the score into wild type (n = 83) or genotype *17 (n = 50). Part (**C**) stratifies the score according to CYP2C19 “normal” (n = 96) or “intermediate” (n = 27) metabolism.

There were no statistically significant differences in the magnitude of the decrease in the ODQ score depending on the *CYP2C19* polymorphisms, as well as the type of CYP2C19 isoenzyme metabolism. All patients showed a significant decrease in the ODQ score after 8 weeks, which was not dependent on *CYP2C19* polymorphisms (Figure 2, Parts A and B) and the type of CYP2C19 metabolism (Figure 2, Part C).

**Figure 3:**
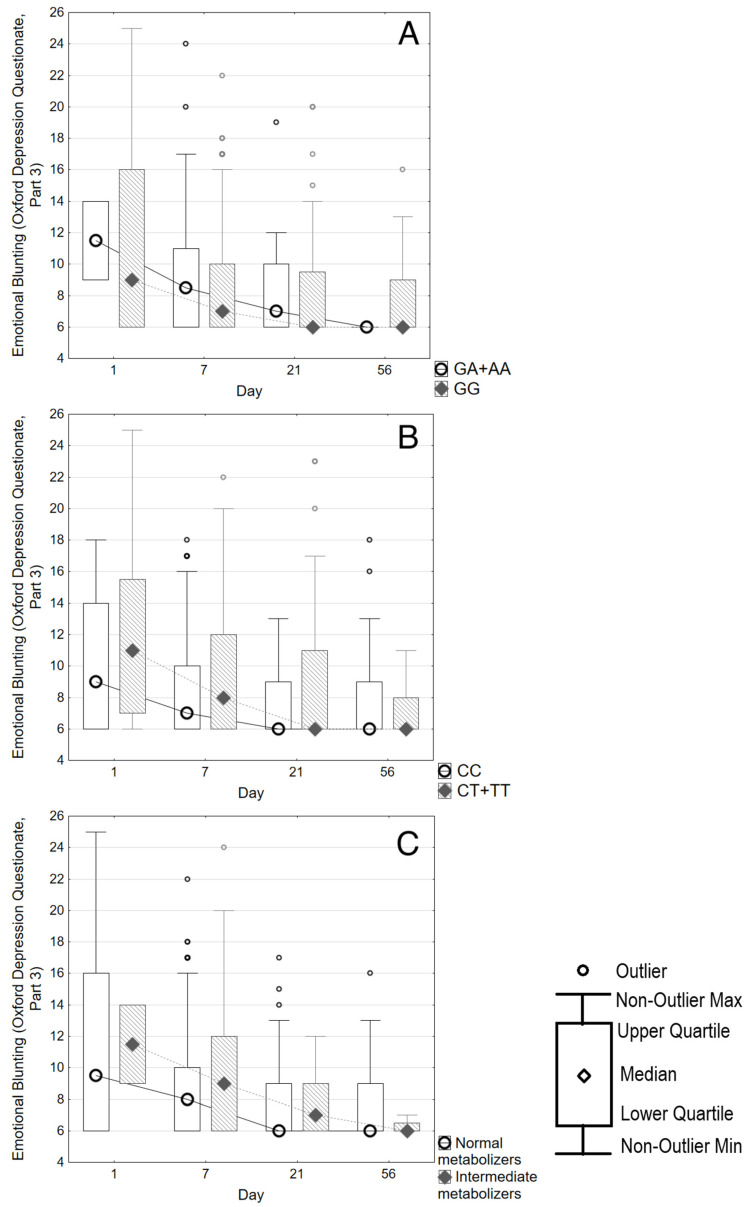
ODQ Part 3 score changes over 8 weeks according to CYP2C19 genotype. Part (**A**) stratifies the score into wild type (n = 105) or genotype CYP2C19*2 GA+AA (n = 28). Part (**B**) stratifies the score into wild type (n = 83) or genotype CYP2C19*17 CT+TT (n = 50). Part (**C**) stratifies the score according to CYP2C19 “normal” (n = 96) or “intermediate” (n = 27) metabolism.

Figure 3 shows the changes in the score of only Part 3 of the ODQ scale. The score remained stable for almost the entire follow-up period and did not differ between carriers of different genotypes of CYP2C19*2 and *17 polymorphisms (Figure 3, Parts A and B). There was also no difference between “normal” and “intermediate” metabolizers of CYP2C19 (Figure 3, Part C).

Sertraline dosages were compared between carriers of the CYP2C19*2 and *17 polymorphisms during this study. The initial dose of sertraline averaged 25[25;25] mg/day and did not differ depending on the polymorphisms of *CYP2C19*. After 1 week, the sertraline dose was 75[75;75] mg/day, and there were no differences depending on the polymorphisms of CYP2C19. After 3 weeks, the sertraline dose in the sample was 100[100;100] mg/day, and significant differences were found for CYP2C19*2, where the dose was slightly lower in GA+AA genotype carriers (100[87.5;100] vs. 100[100;100] mg/day; *p* = 0.048). At week 8, the dose of sertraline was identical to that observed after 3 weeks (100[100;100] mg/day), with no differences depending on *CYP2C19* polymorphisms.

The analysis of the ODQ score revealed a significant difference depending on the prescription of an antipsychotic. Statistically significant differences were also found for the CGI-S, CGI-I, and BDI scales (Table 3).

Antipsychotic administration was associated with greater emotional blunting at inclusion, after 1 week and 3 weeks, but not after 8 weeks. The administration of mood stabilizers, anxiolytics, and anticholinergic drugs was not associated with different ODQ scores.

Analysis of the values of other psychometric scales confirmed that the prescription of antipsychotics in our sample was associated with both greater severity of the condition and less pronounced improvement (CGI-I). At the 8-week examination, patients who were prescribed antipsychotics had significantly higher BDI scores (*p* = 0.01).

We additionally assessed the ODQ score depending on patients’ complaints of sedation. Table 4 shows the results of the analysis. The ODQ score differed significantly depending on the presence of complaints of sedation at 1 and 8 weeks of observation. However, the score for Part 3 of the ODQ did not differ significantly between the selected subgroups of patients at any time during the observation period.

## 4. Discussion

We conducted a study of emotional blunting changes in adolescents with a depressive episode and suicidal intention who were prescribed sertraline. According to our data, this is the first study of a large sample of adolescents with a depressive episode in which emotional blunting was assessed using the ODQ-26. We were able to establish that the ODQ score in adolescents with a depressive episode significantly decreased within 8 weeks. At the same time, the ODQ Part 3 score remained stable and did not increase during the observation period.

The ODQ score was the main outcome in our study. We additionally assessed patients’ complaints about sedation, and this allowed us to distinguish emotional blunting from sedation. But we cannot be completely sure that the patient subjectively did not confuse sedation on the background of pharmacotherapy with emotional blunting. Here, our degree of confidence is limited by the accuracy of the ODQ scale questions. Calculations based on the combination of complaints of sedation and ODQ scores showed that although there were significant associations on day 7 and week 8, this does not apply to Part 3 of the ODQ scale. We can conclude that the emotional blunting that patients reported as acquired during psychopharmacotherapy was clearly distinct from complaints of sedation.

The third part of the ODQ-26 scale in our study had no significant effect on the overall score of the questionnaire. The ODQ scale is a useful tool to assess the alleviation of depressive symptoms based on the use of antidepressants. As follows from previously published studies, antidepressant-induced emotional blunting in adolescents is an infrequent phenomenon [13,14]. The advantage of our study compared with previously published works is the large sample of adolescents with a depressive episode.

The ODQ scale has not been validated in adolescents; previously, it was used only in adults. We considered it possible to use it in adolescents, since this scale does not include any symptoms specific to adult patients only. This approach is justified since the ODQ scale has proven its validity in adult patients. Consequently, the results obtained in adolescents can be compared with those obtained in adult patients. A similar example is the PANSS (Positive and Negative Syndrome Scale), which is used unchanged in adults, adolescents, and even children [27]. In our study, we also used other psychometric scales to avoid false impressions of patients’ condition based on ODQ data.

The only statistically significant finding from this study was the association between the prescription of an antipsychotic medication and the ODQ score. This study was conducted under naturalistic conditions, without influencing medical prescriptions. This allowed for an accurate representation of the treatment process for adolescents with depression in a hospital setting. We could not build a multivariate model to clarify the effect of polypharmacy on the degree of emotional blunting due to insufficient sample size.

Patients with greater levels of emotional blunting were more likely to receive an antipsychotic prescription at the time of enrollment in this study. This difference persisted for up to three weeks after enrollment but was no longer significant at the eight-week follow-up.

In this case, the apathy of patients was present prior to the initiation of pharmacotherapy and was assessed using the ODQ-20 scale (without considering the effects of the antidepressant medication). However, as the difference between the two groups was no longer significant after 8 weeks of follow-up, it is not possible to draw any conclusions regarding the effect of antipsychotic medication on maintaining emotional blunting in patients. The use of antipsychotics, including those from generation 2, has been linked to a risk of emotional blunting [28]. Antipsychotics block dopamine receptors, and an intersection with the likely effect of antidepressants on emotional blunting can be assumed [11]. The development of SSRI-induced apathy is associated with the effect of antidepressants on the frontal cortex [9,10]. SSRIs increase serotonin levels, resulting in increased activity of GABA-ergic interneurons. In turn, this reduces the levels of dopamine and norepinephrine, leading to the development of emotional blunting [10,11]. Dopamine plays a key role in motivation and internal reward processes, so a decrease in the activity of dopaminergic neurons can lead to symptoms of emotional blunting [11]. In our study, it was not confirmed that the simultaneous use of sertraline and an antipsychotic leads to a greater degree of emotional blunting. Although among patients who received additional antipsychotic medication, the severity of depression remained higher at week 8 of observation (according to the BDI score), we cannot say that the prescription of antipsychotic medication caused the depression to worsen. On the contrary, antipsychotics were more often prescribed to patients whose depression was significantly more severe at the time of hospitalization (detailed in Table 3). However, there was a decrease in the average BDI score during the observation period, while there was no increase in the ODQ Part 3 score. Therefore, we can consider the prescription of antipsychotics not as a causal factor in emotional blunting. It is likely that the prescription of antipsychotics was indeed associated with the greater severity of the patients’ mental state. Subsequently, the use of antipsychotics did not affect the maintenance of emotional blunting.

Patient complaints of apathy in the first weeks of taking an antidepressant, according to Peters et al. (2022), should be considered as a residual symptom of the underlying disease [5]. The Peters et al. (2022) [5] study did not use the ODQ; emotional blunting was assessed on a single point of the Montgomery–Åsberg Depression Rating Scale, which does not allow for a full comparison with our results. But the results are consistent in that by the 8th week of therapy, the number of complaints of emotional blunting significantly decreased [5]. Thus, our results in adolescents who were prescribed sertraline were similar to those in adult patients with a depressive episode. Emotional blunting in our sample steadily decreased in all patients by the 8th week of follow-up (confirmed by both the ODQ and the CGI, BDI). It can be concluded that severe emotional blunting is not a common adverse reaction to sertraline in adolescents with a depressive episode.

In our study, we also assessed the contribution of pharmacogenetic factors to the severity of emotional blunting when taking sertraline. Our study did not reveal significant associations of the ODQ-26 score with the carrier status of *CYP2C19* polymorphisms, as well as the type of CYP2C19 metabolism. At the time of inclusion in this study, the “intermediate” CYP2C19 metabolizers had a significantly higher ODQ-20 score (Table 2), but this was not associated with taking sertraline. Subsequently, the difference was eliminated. Thus, our study yielded negative results. Even the differences we found in the daily dose of sertraline depending on the carrier status of CYP2C19*2 at week 3 of follow-up did not significantly contribute to our results. The ODQ score values did not differ between the carriers of CYP2C19*2 and the “wild” genotype. Although the effect of sertraline on the degree of emotional blunting is dose-dependent, we did not observe this effect in our study.

We recognize that our study has methodological limitations. Specifically, these include a small sample size—including a small number of “ultra-rapid” and “slow” CYP2C19 metabolizers—and a limited follow-up period. It is possible that the increase in emotional blunting in adolescents will be observed beyond the 8-week treatment period. This calls for further studies to refine our findings.

The pharmacogenetics of SSRI safety in adolescents has not been sufficiently studied today compared with adults. But the results obtained so far indicate that it is impractical to extrapolate personalization algorithms developed on adults to adolescents [18]. New associative research is required to develop algorithms for personalizing the administration of antidepressants to adolescents. In this study, we found that there are no significant associations between *CYP2C19* polymorphisms and emotional blunting in adolescents with a depressive episode. We cannot compare our results with other studies, including those on adult patients, since no similar studies have been conducted before. However, the negative result of our study is consistent with the fact that the prognostic role of pharmacogenetic testing for the safety of antidepressants in adolescents is less significant than in adults, or paradoxical [17,18].

It is important to note the gender imbalance in our study. Women were in the majority, which may have influenced the results. There is evidence that men and women respond differently to antidepressants. Women generally tolerate SSRIs better than men [29]. In particular, men reported tachycardia more frequently than women when taking nortriptyline [30]. In the study by Marasine et al. (2020), non-compliance was slightly more common among women than among men [31]. Most studies focus on gender differences in relation to menopause, the safety of antidepressants during pregnancy, and the assessment of adverse reactions affecting the reproductive system [32,33]. A large-scale meta-analysis by Pillinger et al. (2025) found no gender differences [34].

These differences are primarily due to the unique characteristics of the female body: a tendency to accumulate antidepressants in adipose tissue, hormonal fluctuations throughout the month, differences in the expression of liver isoenzymes, and, as a result, varying rates of drug metabolism [35]. However, there is no convincing evidence that gender affects CYP2C19 expression; therefore, differences in the rate of metabolism between boys and girls are unlikely for sertraline [36].

Overall, our study cannot currently serve as evidence that sertraline treatment alters ODQ scores in adolescents. This indirectly suggests that emotional blunting during antidepressant treatment in adolescents is less pronounced than the perceived improvement in their condition. Similarly, pharmacogenetic testing also did not demonstrate its significance for predicting ODQ scores in adolescents with a depressive episode. However, further studies with a larger sample size are needed, as pharmacogenetic testing for predicting the safety of antidepressants in adolescents has not been sufficiently studied.

### Limitations

First of all, we must repeat that the ODQ scale has not yet been validated in adolescents. However, we did not consider this to be an obstacle to carrying out our research, as there is currently no other tool for measuring emotional blunting in adolescents. The reliability of the data obtained is also limited by the fact that emotional blunting is based on patients’ self-assessment, and judgments are subjective. It is difficult to distinguish emotional blunting associated with antidepressant use from nonspecific side effects of the drugs or clinical improvement. In the present study, the follow-up period is limited to 8 weeks, which makes it impossible to assess the long-term effects of taking sertraline. The monocentric nature of this study and the absence of a control group or comparison with other antidepressants significantly limit the possibility of extrapolating the results obtained. Our study had a high patient dropout rate, which resulted in a small sample size and low power. It is worth noting the predominance of girls among the patients, which also makes it difficult to extrapolate the results to the general population. Within this study, we conducted pharmacogenetic testing only of *CYP2C19* polymorphisms, though we plan to expand the genetic panel in the future. The naturalistic design of this study did not exclude the effect of additional pharmacotherapy. In addition, due to the multiple comparisons in our study, there is a high probability of type 2 error (false negative). This should be considered when replicating this study on a larger sample.

## 5. Conclusions

In adolescents with a depressive episode and suicidal intentions, there was no significant effect of taking sertraline on the increasing of emotional blunting according to the ODQ score over 8 weeks. According to our data, this is the first study of adolescents with a depressive episode using the ODQ scale to assess emotional blunting while taking an SSRI. Our conclusions are based on the fact that the ODQ Part 3 score remained stable over the observation period.

Our study found no significant associations of the carrier status of *CYP2C19* polymorphisms for ODQ score changes. Thus, the pharmacogenetic part of our study also showed a negative result. This highlights the importance of conducting new associative studies to identify significant pharmacogenetic factors in the efficacy and safety of antidepressants in adolescents with a depressive episode.

## Figures and Tables

**Table 1:** Baseline characteristics of the patients who completed this study.

Variable	Participants Who Enrolled in This Study (n = 133)	Participants Who Completed This Study (n = 88)	*p*
Age	14[13;16]	15[14;16]	0.8716
Height, m	1.63[1.58;1.687]	1.62[1.58;1;69]	0.6107
Weight, kg	54.0[47.0;62.1]	53.5[48.45;62.9]	0.5852
Body mass index	19.9[18.1;22.3]	20.2[18.4;22.3]	0.9406
Females	89.5%	88.60%	0.8293
Total number of hospitalizations (including the current one)	1[1;1]	1[1;1]	0.7393
Age of onset of symptoms of mental disorder	13[12;14]	13[12;14]	0.7008
Suicidal thoughts	93.2%	97.70%	0.2067
A history of NSSI	91.0%	97.70%	0.0504
Age of occurrence of NSSI	13[12;14]	14[13;14]	0.8004
Adolescents who attempted suicide	39.8%	44.30%	0.5775
Age at the start of taking antidepressants	14[13;16]	15[14;16]	0.7814
Duration of mental disorder before inclusion in this study	16[8;27]	18[8;27]	0.8157

Notes: Quantitative variables are presented with medians and 95% confidence intervals; NSSI—non-suicidal self-injury.

**Table 2:** Oxford Depression Questionnaire (ODQ) score values depending on the carrier status of polymorphic variants of CYP2C19*2, CYP2C19*17, and type of metabolism of CYP2C19.

Moment of Inclusion	All Patients (n = 133)	CYP2C19*2		*p*	CYP2C19*17		*p*	CYP2C19 Metabolism		*p*
		GG (n = 105)	GA+AA (n = 28)		CC (n = 83)	CT+TT (n = 50)		“Normal” (n = 96)	“Intermediate” (n = 27)	
Overall ODQ score	65[50;79]	64[48;78]	67.5[58;84]	0.146	65[49;79]	63.5[50;80]	0.678	64[49;78.5]	69[59;84]	0.078
ODQ, Parts 1–2 score	64[49;77]	63[44;76]	67.5[58;84]	0.067	65[49;77]	63[52;78]	0.772	63[44.5;76]	69[59;84]	0.028
ODQ, Part 3 score (n = 21) *	9[6;14]	9[6;16]	11.5[9;14]	0.686	9[6;14]	11[7;15.5]	0.645	9.5[6;16]	11.5[9;14]	0.758
CGI-S	4[3;4]	4[3;4]	4[3.5;5]	0.042	4[3;4]	4[3;5]	0.762	4[3;4]	4[3;5]	0.071
BDI	23[12;34]	21[11;33]	26[18;36]	0.067	23[12;33]	21[11;35]	0.939	21[11;32.5]	25[18;37]	0.078
**Examination After 1 Week**	**All Patients (n = 133)**	CYP2C19*2		***p***	CYP2C19*17		***p***	**CYP2C19 Metabolism**		***p***
		**GG (n = 105)**	**GA+AA (n = 28)**		**CC (n = 83)**	**CT+TT (n = 50)**		**“Normal” (n = 96)**	**“Intermediate” (n = 27)**	
Overall ODQ score	65[50;79]	62[50;78]	66[55.5;79.5]	0.332	63[47;79]	65.5[53;79]	0.726	63.5[50;79]	66[54;80]	0.275
ODQ, Parts 1–2 score	54[41;69]	53[40;69]	56[47;67.5]	0.372	54[40;68] n = 83	54[42;69] n = 50	0.866	53.5[40;69]	57[46;69]	0.341
ODQ, Part 3 score	8[6;10]	7[6;10]	8.5[6;11]	0.280	7[6;10] n = 83	8[6;12] n = 50	0.615	8[6;10]	9[6;12]	0.379
CGI-S	3[2;4]	3[2;4]	3.5[3;4]	0.184	3[2;4]	3[2;4]	0.341	3[2;4]	3[3;4]	0.206
CGI-I	3[2;3]	3[2;3]	3[3;3.5]	0.148	3[2;3]	3[3;3]	0.596	3[2;3]	3[3;4]	0.131
BDI	12[5;21]	12[5;21]	15[7;20.5]	0.280	12[4;20]	12.5[7;22]	0.480	12[4.5;20]	14[6;21]	0.329
**Examination After 3 Weeks**	**All Patients (n = 112)**	CYP2C19*2		***p***	CYP2C19*17		***p***	**CYP2C19 Metabolism**		***p***
		**GG (n = 85)**	**GA+AA (n = 27)**		**CC (n = 69)**	**CT+TT (n = 43)**		**“Normal” (n = 78)**	**“Intermediate” (n = 26)**	
Overall ODQ score	48[39;62.5]	47[39;63]	50[43;58]	0.42	48[39;63]	46[39;59]	0.734	47.5[39;64]	49[43;56]	0.6300
ODQ, Parts 1–2 score	41[32.5;54]	41[32;55]	42[34;48]	0.522	41.5[33;54]	39[32;50]	0.6251	41[32;56]	42[34;48]	0.7174
ODQ, Part 3 score	6[6;9.5]	6[6;9]	7[6;10]	0.235	6[6;9]	6[6;11]	0.4744	6[6;9]	7[6;9]	0.2603
CGI-S	3[2;3]	2[2;3]	3[2;3]	0.945	3[2;3]	3[2;3]	0.891	2.5[2;3]	3[2;3]	0.9020
CGI-I	2[2;2]	2[2;2]	2[2;2]	0.319	2[2;2]	2[2;2]	0.4968	2[2;2]	2[2;2]	0.2297
BDI	7[3;14]	6[3;14]	8[3;13]	0.797	8[4;13]	6[2;14]	0.6635	6.5[3;15]	8[3;13]	0.9375
**Examination After 8 Weeks**	**All Patients (n = 88)**	CYP2C19*2		***p***	CYP2C19*17		***p***	**CYP2C19 Metabolism**		***p***
		**GG (n = 68)**	**GA+AA (n = 20)**		**CC (n = 55)**	**CT+TT (n = 33)**		**“Normal” (n = 64)**	**“Intermediate” (n = 19)**	
Overall ODQ score	38.5[32.5;56.5]	38.5[32.5;57]	37.5[32.5;51.5]	0.7332	38[32;56]	39[34;57]	0.3449	38[32;56]	39[32;56]	0.9871
ODQ, Parts 1–2 score	32[26.5;49]	32[26.5;49]	31.5[26.5;45.5]	0.7783	32[26;48]	33[28;50]	0.2987	32[26;46.5]	33[26;50]	0.9186
ODQ, Part 3 score	6[6;8.5]	6[6;9]	6[6;6.5]	0.4794	6[6;9]	6[6;8]	0.8103	6[6;8.5]	6[6;7]	0.6476
CGI-S	1[1;2]	1[1;3]	1[1;1.5]	0.4741	1[1;2]	1[1;3]	0.5960	1[1;2]	1[1;2]	0.6305
CGI-I	1[1;2]	1[1;2]	1[1;1.5]	0.3116	1[1;2]	1[1;2]	0.4631	1[1;2]	1[1;2]	0.4417
BDI	6[4;13]	6[4;13]	6[5;10]	0.8521	6[4;10]	7[;4;13]	0.6540	6[4;12]	6[4;10]	0.7936

Notes: Me—median; Q—quartile; ODQ—Oxford Depression Questionnaire; CGI-S—Clinical Global Impression–Severity; CGI-I—Clinical Global Impression–Improvement; BDI—Beck Depression Inventory. * At the time of inclusion in this study, the ODQ Part 3 score was assessed in only 21 adolescents who had taken SSRIs prior to hospitalization.

**Table 3:** The value of the Oxford Depression Questionnaire score and other psychometric scales, depending on the prescribing of additional pharmacotherapy.

Moment of Inclusion	Antipsychotic		*p*	Mood Stabilizer		*p*	Anxiolytic		*p*	Anticholinergic Drug		*p*
	Yes (n = 70)	No (n = 63)		Yes (n = 17)	No (n = 116)		Yes (n = 64)	No (n = 69)		Yes (n = 20)	No (n = 113)	
Overall ODQ score	73[56;83]	59[44;71]	0.0006	61[42;69]	65.5[50.5;79.5]	0.389	62.5[48.5;80.5]	67[54;78]	0.433	65[49.5;85.5]	65[50;77]	0.5380
Parts 1–2 score	70[53;79]	59[38;71]	0.0023	58[42;67]	65[49.5;78]	0.206	62.5[43.5;80.5]	65[50;76]	0.675	65[49;84]	64[50;76]	0.5631
Part 3 score (n = 21)	13[9;18]	7.5[6;10]	0.072	9[6;13]	9.5[6.5;15]	0.780	9[6;14]	10[6;17]	0.654	9[6;13]	9.5[6;16]	0.6691
CGI-S	4[3;5]	4[3;4]	0.0049	4[3;4]	4[3;4]	0.657	4[3;4]	4[3;4]	0.410	4[3.5;5]	4[3;4]	0.2299
BDI	28.5[16;38]	18[8;28]	0.0007	20[15;28]	23[12;34.5]	0.514	23[15;33]	22[11;35]	0.561	31[14;37.5]	21[12;33]	0.1742
**Examination After 1 Week**	**Antipsychotic**		***p***	**Mood Stabilizer**		***p***	**Anxiolytic**		***p***	**Anticholinergic Drug**		***p***
	**Yes (n = 70)**	**No (n = 63)**		**Yes (n = 17)**	**No (n = 116)**		**Yes (n = 64)**	**No (n = 69)**		**Yes (n = 20)**	**No (n = 113)**	
Overall ODQ score	68[54;81]	57[43;73]	0.0075	65[50;69]	65.5[51;79]	0.8640	59.5[51;77]	66[50;79]	0.7480	67.5[57.5;80]	65[47;78]	0.2850
ODQ, Parts 1–2 score	58[42;73]	48[7;65]	0.0114	52[42;61]	54.5[40.5;69]	0.8177	55[41;69]	52[42;68]	0.8947	57.5[50.5;70.5]	52[40;68]	0.1606
ODQ, Part 3 score	8[6;11]	7[6;10]	0.5427	8[6;10]	7[6;10]	0.4639	7[6;10]	8[6;10]	0.6486	6.5[6;9]	8[6;10]	0.3148
CGI-S	4[3;4]	3[2;4]	0.0051	3[2;4]	3[2;4]	0.6224	3[2;4]	3[3;4]	0.2640	4[3;4]	3[2;4]	0.2883
CGI-I	3[3;3]	3[2;3]	0.3106	3[2;3]	3[2;3]	0.8281	3[2;3]	3[3;3]	0.9516	3[3;3]	3[2;3]	0.6915
BDI	14[7;24]	11[3;18]	0.0140	11[6;16]	13[5;21]	0.5813	13.5[5;21.5]	11[5;18]	0.5640	13.5[8.5;22]	12[5;20]	0.3461
**Examination After 3 Weeks**	**Antipsychotic**		***p***	**Mood Stabilizer**		***p***	**Anxiolytic**		***p***	**Anticholinergic Drug**		***p***
	**Yes (n = 64)**	**No (n = 48)**		**Yes (n = 15)**	**No (n = 97)**		**Yes (n = 50)**	**No (n = 62)**		**Yes (n = 18)**	**No (n = 94)**	
Overall ODQ score	50.5[41.5;68]	45.5[36;54]	0.0150	51[47;58]	47[39;63]	0.211	47[39;66]	48.5[39;58]	0.9883	48.5[42;66]	48[39;59]	0.4864
ODQ, Parts 1–2 score	42[34.5;60]	39[29;46.5]	0.0341	42[39;60]	41[32;53]	0.742	40.5[32;59]	41[32;50]	0.7684	42[36;60]	41[32;53]	0.5475
ODQ, Part 3 score	6[6;10]	6[6;8]	0.1741	7[6;11]	6[6;9]	0.248	6[6;9]	6[6;10]	0.8726	6[6;10]	6[6;9]	0.4572
CGI-S	3[2;3]	2[1;3]	0.0001	3[2;3]	3[2;3]	0.852	2.5[1;3]	3[2;3]	0.8269	3[2;3]	2.5[2;3]	0.4199
CGI-I	2[2;3]	2[1;2]	0.0014	2[2;2]	2[2;2]	0.933	2[2;3]	2[2;2]	0.8588	2[2;2]	2[2;2]	0.3229
BDI	9[3.5;18]	5.5[0.5;12.5]	0.0178	7[3;13]	7[3;14]	0.793	8.5[2;17]	6.5[3;12]	0.4430	6.5[4;18]	7[2;14]	0.4668
**Examination After 8 Weeks**	**Antipsychotic**		***p***	**Mood Stabilizer**		***p***	**Anxiolytic**		***p***	**Anticholinergic Drug**		***p***
	**Yes (n = 53)**	**No (n = 35)**		**Yes (n = 13)**	**No (n = 75)**		**Yes (n = 37)**	**No (n = 51)**		**Yes (n = 16)**	**No (n = 72)**	
Overall ODQ score	40[33;60]	36[32;49]	0.0920	41[29;51]	38[32;57]	0.5686	39[34;67]	38[32;66]	0.3812	36.5[32.5;60.5]	38.5[32.5;56.5]	0.9872
ODQ, Parts 1–2 score	33[27;52]	30[26;40]	0.1111	34[29;47]	32[26;50]	0.6840	33[28;56]	32[26;47]	0.3412	30.5[26.5;53.5]	32[26.5;47.5]	0.9274
ODQ, Part 3 score	6[6;9]	6[6;9]	0.3203	7[6;9]	6[6;8]	0.1822	6[6;10]	6[6;8]	0.5116	6[6;6]	6[6;9]	0.3202
CGI-S	1[1;3]	1[1;2]	0.1596	1[1;3]	1[1;2]	0.8302	1[1;2]	1[1;2]	0.9861	1.5[1;3]	1[1;2]	0.3078
CGI-I	1[1;2]	1[1;2]	0.1444	1[1;2]	1[1;2]	0.9809	1[1;2]	1[1;2]	0.9861	1.5[1;2.5]	1[1;2]	0.2675
BDI	7[5;16]	5[2;10]	0.0105	7[4;16]	6[4;12]	0.6933	7[4;12]	6[4;13]	0.4807	6.5[5.5;17]	6[4;12]	0.1777

Notes: ODQ—Oxford Depression Questionnaire; CGI-S—Clinical Global Impression–Severity; CGI-I—Clinical Global Impression–Improvement; BDI—Beck Depression Inventory.

**Table 4:** The value of the Oxford Depression Questionnaire score depending on the presence of sedation complaints.

ODQ Scores	Sedation After 1 Week		*p*
	Yes (n = 61)	No (n = 72)	
Overall ODQ score	72[55;82]	60[46.5;71.5]	0.011
ODQ, Parts 1–2 score	64[46;74]	51[39.5;61.5]	0.008
ODQ, Part 3 score	8[6;10]	7[6;10]	0.626
ODQ Scores	Sedation After 3 Weeks		*p*
	Yes (n = 35)	No (n = 77)	
Overall ODQ score	51[39;68]	48[41;58]	0.536
ODQ, Parts 1–2 score	42[33;60]	41[33;50]	0.424
ODQ, Part 3 score	6[6;10]	6[6;10]	0.535
ODQ Scores	Sedation After 8 Weeks		*p*
	Yes (n = 7)	No (n = 81)	
Overall ODQ score	65[40;81]	38[32;56]	0.057
ODQ, Parts 1–2 score	57[347;64]	32[26;46]	0.047
ODQ, Part 3 score	6[6;12]	6[6;8]	0.525

Notes: ODQ—Oxford Depression Questionnaire.

## Data Availability

The data presented in this study are available upon request from the corresponding author.

## Abbreviations

The following abbreviations were used in this manuscript:

BMI	Body mass index
ODQ	Oxford Depression Questionnaire
